# Tailoring Strength and Corrosion Resistance in Al–Zn–Mg–Cu Alloys by Total (Zn + Mg) Content and Multi-Directional Forging Process

**DOI:** 10.3390/ma18194476

**Published:** 2025-09-25

**Authors:** Junfu Lin, Tangjian Liu, Mingdong Wu, Shuo Yuan, Zeyu Li, Yang Huang, Xiao Yin, Lanping Huang, Wensheng Liu, Daihong Xiao

**Affiliations:** National Key Laboratory of Science and Technology on High-Strength Structural Materials, Central South University, Changsha 410083, China; 13737953027@163.com (J.L.); liutangjian1998@163.com (T.L.); mmingshiddong@csu.edu.cn (M.W.); shuoyuan@csu.edu.cn (S.Y.); 16620176815@163.com (Z.L.); youngh@csu.edu.cn (Y.H.); 213301042@csu.edu.cn (X.Y.); christie@csu.edu.cn (L.H.); liuwensheng@csu.edu.cn (W.L.)

**Keywords:** Al–Zn–Mg–Cu alloys, microstructure, mechanical properties, corrosion resistance, mortise and tenon nested grain structure

## Abstract

The effects of (Zn + Mg) total content (9.6–11.7 wt.%) combined with multi-directional forging (MDF) on the microstructure and properties of high-strength Al–Zn–Mg–Cu alloys were systematically investigated. Our results demonstrate that the alloy obtains significant grain refinement, which is attributed to the dynamic recrystallization in the MDF process. Specifically, Al-8.6Zn-1.55Mg-1.9Cu-0.11Zr (Zn + Mg = 10.15 wt.%) obtains the maximum recrystallization ratio (51.8%) and the weakest texture strength, and also forms the mortise and tenon nested grain structure. Increasing the total (Zn + Mg) content can achieve significant performance enhancement, which is attributed to the refinement of the η′ phase; however, a higher total (Zn + Mg) content will lead to the continuous distribution of coarse η-MgZn_2_ phases formed along the grain boundary, accompanied by the broadening of precipitate-free precipitation zones (PFZs). Compared with other alloys, Al-8.6Zn-1.55Mg-1.9Cu-0.11Zr (Zn + Mg = 10.15 wt.%) maintains high strength while ensuring desirable plasticity due to its mortise and tenon nested grain structure. In addition, its desirable grain boundary precipitation behavior makes it exhibit the best corrosion resistance. These findings indicate that maintaining the total (Zn + Mg) content around 10 wt.% achieves a balance between strength and corrosion resistance, offering a theoretical foundation for the design of high-strength and corrosion-resistant Al–Zn–Mg–Cu alloys.

## 1. Introduction

As a representative of high-strength structural materials, Al–Zn–Mg–Cu alloys are widely favored in construction, automotive, aerospace, and rail transit applications due to their excellent specific strength, formability, and weldability [1,2,3]. Extensive research efforts have been devoted to enhancing corrosion resistance while simultaneously improving strength, primarily through the optimization of alloying element content (e.g., Zn and Mg) and thermomechanical processing techniques (e.g., extrusion, rolling, and forging) [4,5,6]. Among these techniques, severe plastic deformation (SPD) has gained widespread attention as an effective means of achieving grain refinement and strengthening (e.g., high-pressure torsion, accumulative roll bonding, and multi-directional forging) [7,8,9].

Multi-directional forging (MDF) can achieve a refined microstructure and superior mechanical performance, which is applied in diverse metallic systems [10,11]. In this technique, the material undergoes a forging process in its three orthogonal directions. During the MDF process, the fine grains inside the alloy grains will gradually increase with the increase in the total strain, resulting in an increase in dislocation density and lattice distortion. These defects promote the dissolution of a coarse second phase and enhance the driving force of aging precipitation. The increase in fine grains and the refinement of aging precipitates will promote the strength of the alloy [12,13,14]. In addition, MDF will not only increase the fine grains in the alloy, but eventually leads to the coexistence of fine grains and coarse grains to form a multi-scale grain structure. This grain structure inhibits grain boundary slip and allows synergistic deformation to improve mechanical properties [15,16]. Several studies have been carried out on grain refinement and improving the mechanical properties of the alloy by MDF. MIURA H et al. [17] applied multi-pass MDF to AZ61 magnesium alloy to refine the grain size of the alloy to 0.8 μm, and the tensile strength reached 440 MPa, with room-temperature superplasticity. Wang et al. [18] conducted MDF treatment on 2A14 aluminum alloy, showing that as the cumulative strain increases from 0.4 to 7.2, the dislocation density and average grain size first increase and then decrease. It has also been reported that an increase in the cumulative deformation of MDF results in more severe lattice distortion and defect formation, which is more favorable for the dissolution of coarse secondary-phase particles. This, in turn, enhances the driving force for aging precipitation, thereby improving the mechanical strength of the alloy [16].

In recent years, research on Al–Zn–Mg–Cu alloys has increasingly focused on the highly alloyed compositions, which can provide superior mechanical strength [19,20]. Zn concentration is a pivotal factor governing the precipitation kinetics of strengthening phases. Elevated Zn levels accelerate both the rate and volume of phase formation, resulting in improved strength [21]. Similarly, a higher Mg content contributes to increased hardness and strength. Reduced precipitate dimensions at grain boundaries occur with excessive Mg, coupled with significantly raised area fractions that foster intergranular fracture and degrade toughness [22]. In addition, elevated Zn and Mg contents promote the formation of MgZn_2_ phases, which lowers the passivation potential and increases the corrosion susceptibility of the alloy. On the other hand, a higher Cu content raises the passivation potential and reduces the potential difference between grain boundary precipitates (GBPs) and the matrix. These alloying elements also influence the continuity of GBPs and the width of the precipitate-free zone (PFZ), thereby further affecting the corrosion behavior. Therefore, examining the effects of main element content on the microstructure and properties of materials is a significant area of research [23,24]. According to Zou et al. [25], dominant precipitation shifts to the η′ phase above a Zn/Mg ratio of 4.44 (wt.%). Nevertheless, elevating this ratio to 11 (wt.%) induces coarsening of the precipitates and a decline in mechanical properties [26]. For Al–Zn–Mg–Cu alloys, the impact of total (Zn + Mg) content on their microstructure, mechanical properties, and corrosion durability is an area that has been insufficiently researched.

This research examined Al–Zn–Mg–Cu alloys with various total (Zn + Mg) concentrations. The processing techniques employed included MDF, solid-solution treatment, and two-stage artificial aging. Subsequently, the effects of grain structure and aging precipitation characteristics on mechanical properties and corrosion resistance were analyzed. The corrosion resistance of the alloys was assessed via electrochemical testing. These results establish a theoretical basis for optimizing the composition and processing of high-strength, corrosion-resistant Al–Zn–Mg–Cu alloys.

## 2. Experimental Section

### 2.1. Materials Preparation

**Figure 1:**
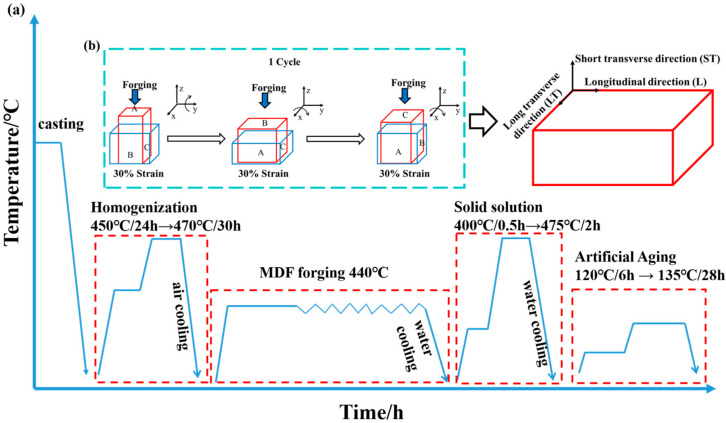
(**a**) Heat treatment process flow chart; (**b**) deformation process flow chart.

This research fabricated alloys from highly pure Al, Zn, Mg, master alloys of Al-50Cu and Al-5Zr (wt.%). The melting process was commenced with high-purity aluminum. After the complete melting of the aluminum, Al-20Cu and Al-5Zr master alloys were introduced sequentially, followed by additions of pure Zn and pure Mg. The raw materials were processed in an electric resistance furnace at 760 °C under laboratory air. Following refinement and degassing with Ar and C_2_Cl_6_, the melt was cast in a preheated iron mold at 200 °C. ICP-AES analysis (iCAP 7600, Thermo Fisher Scientific, Waltham, MA, USA) determined ingot compositions (Table 1). The homogenization process involved dual annealing stages: 450 °C/24 h→470 °C/30 h. After homogenization, the ingots were heated to 440 °C for 1 h before forging. The ingot was preheated at 440 °C for 1 h before hot forging. The specific deformation process is illustrated in Figure 1b. In each pass, the sample was successively deformed by 30% (a true strain of ε = 0.03) along each of the three orthogonal axes, maintaining a constant strain rate of 0.03 s^−1^. A 30 min annealing treatment was applied prior to the next pass to relieve stresses, enhance plasticity, and facilitate subsequent deformation. This cycle was repeated for a total of six passes. The forged alloys experienced solution treatment at 475 °C for 2 h, followed by water quenching. Finally, a two-stage aging treatment (120 °C for 6 h + 135 °C for 28 h) was applied to promote the formation of nanoprecipitates.

### 2.2. Mechanical Property Tests

All mechanical property tests and microstructural observations were conducted on samples taken from a section located at 1/4 of the normal direction. Tensile specimens were oriented with their loading axes parallel to the longitudinal direction (L) of the samples (Figure 1b). Uniaxial tensile tests were conducted using an Instron 3369 universal testing machine (Instron, Norwood, MA, USA) at a constant crosshead speed of 2 mm/min. Triplicate specimens were tested for each condition to obtain averaged data for yield strength (YS), ultimate tensile strength (UTS), and elongation (EL).

### 2.3. Microstructure Characterization

Metallographic specimens were prepared via mechanical polishing and Graff Sargent’s etching (3 g CrO_3_ + 16 mL HNO_3_ + 1 mL HF + 83 mL H_2_O). Resultant microstructures underwent characterization via scanning electron microscopy (SEM, TESCAN MIRA4 LMH, Tescan, Brno, Czech Republic) and optical microscopy (OM, RX50M, Ningbo Sunny Instruments, Ningbo, China). Electropolishing using a solution of 10 vol.% HClO_4_ in 90 vol.% C_2_H_5_OH at 15 V effectively removed mechanically deformed layers introduced during sample preparation from the electron backscatter diffraction (EBSD) specimens, and a 0.5 µm step size was applied for EBSD scanning. The EBSD data were processed and analyzed using Channel 5 and Aztec 5.1 software. TEM analysis employed a Talos F200X/Super-X EDS system (Thermo Fisher Scientific, Waltham, MA, USA). Bright-field (BF), high-angle annular dark-field STEM (HAADF) and high-resolution (HR) imaging modes were employed. Sample preparation involved the following: (1) mechanical grinding to a thickness of 50 μm; (2) punching into 3 mm disks; (3) twin-jet polishing at 15 V using HNO_3_:CH_3_OH (3:7) electrolyte. The TEM data were processed and analyzed using Nano Measurer 1.2 software to analyze and count the particle size and number.

### 2.4. Electrochemical Test

Intergranular corrosion (IGC) testing was performed by immersing the samples in a solution containing 10 mL/L H_2_O_2_ and 57 g/L NaCl at 35 ± 2 °C for 6 h (GB/T 7998-2023 [27]). The cross-sectional features of corroded specimens were examined using an optical microscope (OM, RX50M), while IGC resistance was determined through the maximum corrosion depth. Electrochemical experiments utilized a 3.5 wt.% NaCl solution and a CHI660E workstation (CH Instruments Inc., Shanghai, China). This workstation employed a standard three-electrode configuration: a platinum auxiliary electrode, a saturated calomel reference electrode (SCE), and the sample serving as the working electrode. During the experiments, the exposed area of the sample was 1 cm^2^, and the OCP stabilization time was about 180 s. Potentiodynamic polarization scans were acquired by sweeping the potential between −1300 mV and −300 mV at 2 mV/s.

## 3. Results

### 3.1. Microstructure of As-Cast and Forged Alloy

**Figure 2:**
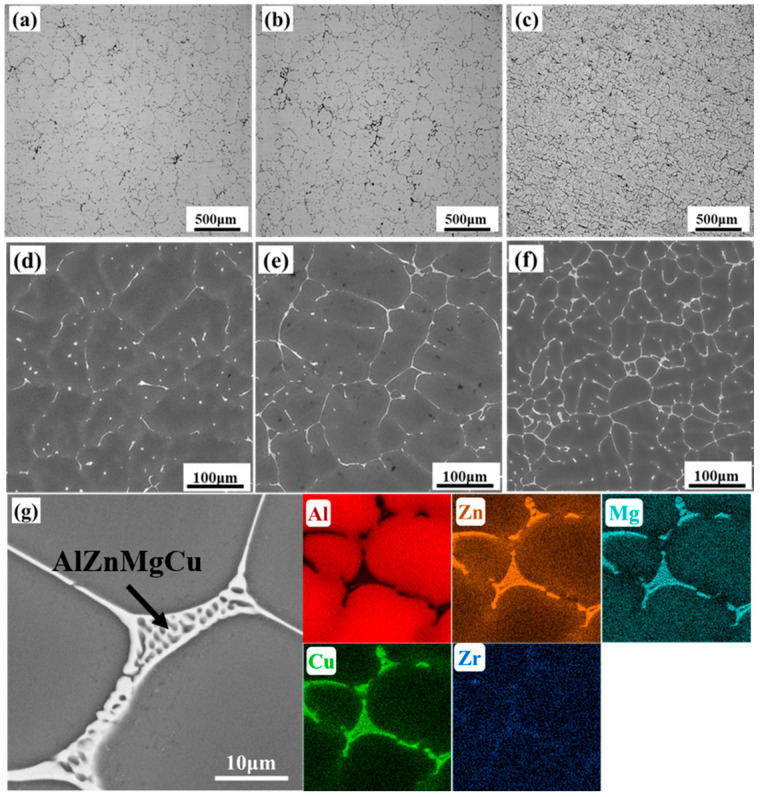
Microstructural characterization of the as-cast alloy: (**a**,**d**) Alloy 1; (**b**,**e**) Alloy 2; (**c**,**f**) Alloy 3; (**a**–**c**) OM image and (**d**–**g**) SEM image and the corresponding EDS image.

The OM micrographs (Figure 2a–c) indicate that dendritic structures in all as-cast alloys are similar and accompanied by coarse non-equilibrium phases. As the total (Zn + Mg) content increases, the dendrite spacing becomes narrower, and the dendritic structure appears more defined, indicating enhanced dendritic segregation (Figure 2d–f). Concurrently, the amount of second-phase particles or precipitates located in the interdendritic regions also increases. The SEM images and corresponding EDS analysis in Figure 2d–g indicate that AlZnMgCu phases in the as-cast alloys can be clearly identified. These phases are characterized by a white network distribution in the interdendritic region and enrichment of Al, Zn, Mg and Cu. It is generally believed that the chemical composition is Mg_32_(AlZn)_49_, Al_2_Mg_3_Zn_3_ and Al_6_CuMg_4_ [28].

**Figure 3:**
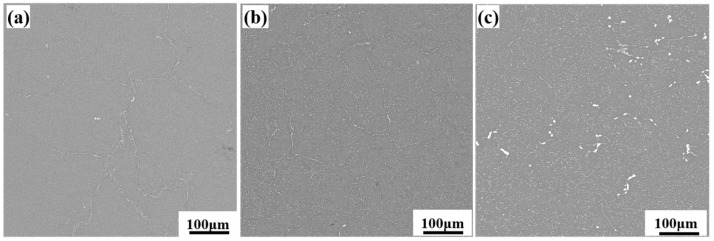
SEM images analysis in L direction: (**a**) Alloy 1; (**b**) Alloy 2; (**c**) Alloy 3.

SEM images of the L-LT surface of the forged alloy after deformation are presented in Figure 3. After MDF, the coarse network secondary phases were significantly fragmented to form the dispersed white second-phase particles in the figure. Moreover, a lower total (Zn + Mg) content correlates with a decreased area fraction of this phase. This phenomenon may be attributed to the limited solid solubility of Zn and Mg in the aluminum matrix. Once the total (Zn + Mg) content exceeds the solubility limit, the excess Zn and Mg tend to precipitate as second-phase particles [29].

### 3.2. Microstructure After Aging Treatment

**Figure 4:**
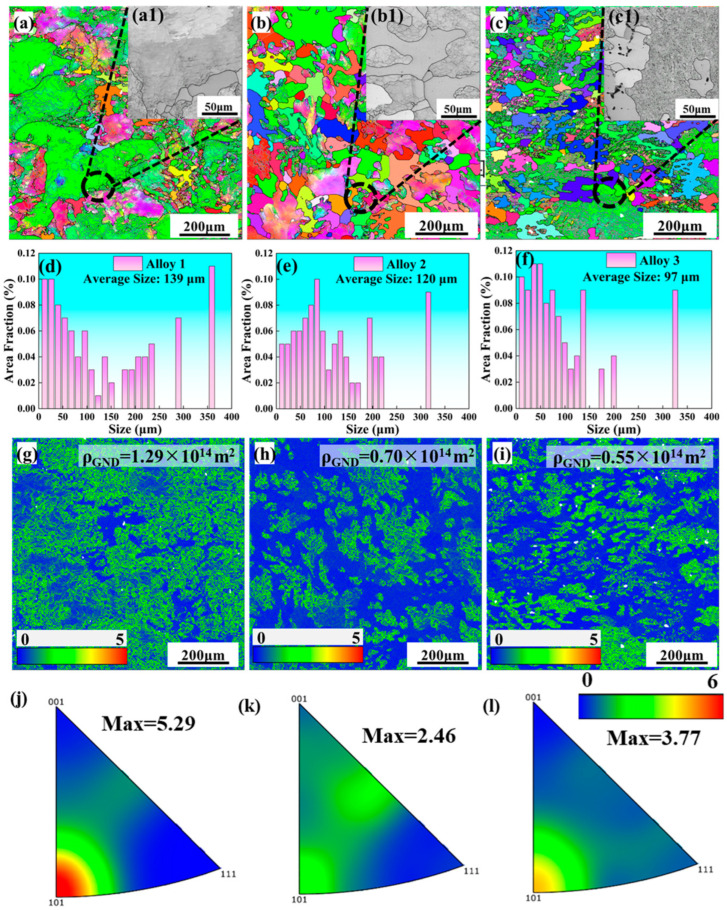
EBSD characterization of the aging-treated alloy: (**a**,**a1**,**d**,**g**,**j**) Alloy 1; (**b**,**b1**,**e**,**h**,**k**) Alloy 2; (**c**,**c1**,**f**,**i**,**l**) Alloy 3; (**a**–**c**,**a1**–**c1**) is the IPF + GB diagram; (**d**–**f**) is the variation trend of the grain size; (**g**–**i**) is the KAM diagram; (**j**–**l**) is the inverse pole figure.

Figure 4 shows the EBSD images of the LT planes of Al–Zn–Mg–Cu alloy after T74 heat treatment, where the black lines represent the grain boundaries with an orientation angle greater than 15°, and the white lines represent the sub-grain boundaries with an orientation angle of 2–15°. As the total (Zn + Mg) content increases, the small-angle sub-grain boundaries increase significantly (Figure 4a–c). In addition, grain refinement is caused by dynamic recrystallization during the MDF process. The grain sizes decrease with the increase in total (Zn + Mg) content, which are 139, 120 and 97 μm, respectively (Figure 4d–f), and the grain orientation is roughly the same. It can be seen from the diagram that, at lower (Zn + Mg) content, the grains of the alloy after T74 treatment are mostly sub-grains. This will lead to a significant reduction in the internal dislocation density (compared to the deformed state); however, it is still higher than the original or recrystallized grains. With the increase in the total (Zn + Mg) content, the proportion of recrystallized grain structure of the alloy grains increases, which will reduce the internal dislocation density. Figure 4g–i present the kernel average misorientation (KAM) maps corresponding to the three alloys. The KAM values, which reflect local lattice distortion, can be quantitatively associated with the density of geometrically necessary dislocations (GNDs) [30]:

(1) $$\rho_{GND}=\frac{2<\theta >}{bd}$$

where $\rho_{GND}$ is the dislocation density (m^−2^), θ is the average local misorientation, b = 0.286 nm is the Burgers vector, and d = 0.15 μm is the step size. The GNDs of Alloys 1, 2 and 3 are 1.29 × 10^14^ m^−2^, 0.70 × 10^14^ m^−2^ and 0.55 × 10^14^ m^−2^, respectively.

Observation of the local grain structure reveals that Alloy 2 exhibits a mortise–tenon grain structure (Figure 4(a1–c1)). This grain structure is postulated to enhance mechanical properties by improving structural stability [15]. Inverse pole figure (IPF) maps illustrating aged samples with different total (Zn + Mg) contents are displayed in Figure 4j–l. It can be observed that the grain orientation in Alloy 1 and Alloy 3 is predominantly concentrated along the (101)Al direction. In contrast, Alloy 2 exhibits a more random orientation distribution. As the total (Zn + Mg) content increases, the overall texture intensity gradually decreases, which ultimately results in a more random grain orientation.

**Figure 5:**
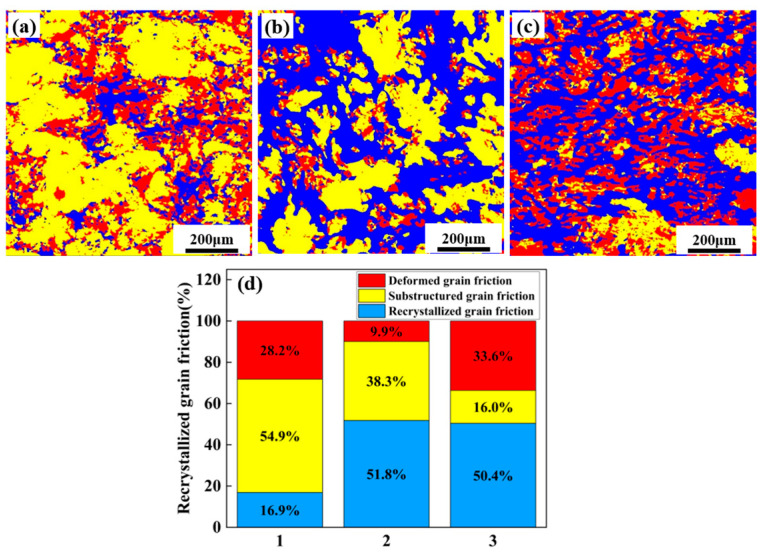
Recrystallization diagram of three alloys: (**a**) Alloy 1; (**b**) Alloy 2; (**c**) Alloy 3; (**d**) Recrystallization area proportion of alloys.

Figure 5 shows the recrystallization behavior of alloys. The blue, yellow and red in the diagram correspond to the recrystallized grains, sub-grains and deformed grains, respectively. Figure 5d is the corresponding proportion diagram. With increasing total (Zn + Mg) content, the degree of recrystallization gradually increases. Alloy 1 is dominated by sub-grains, accounting for 54.9%. Alloys 2 and 3 are mainly composed of recrystallized grains, accounting for 51.8% and 50.4%, respectively.

### 3.3. Precipitation Behavior

**Figure 6:**
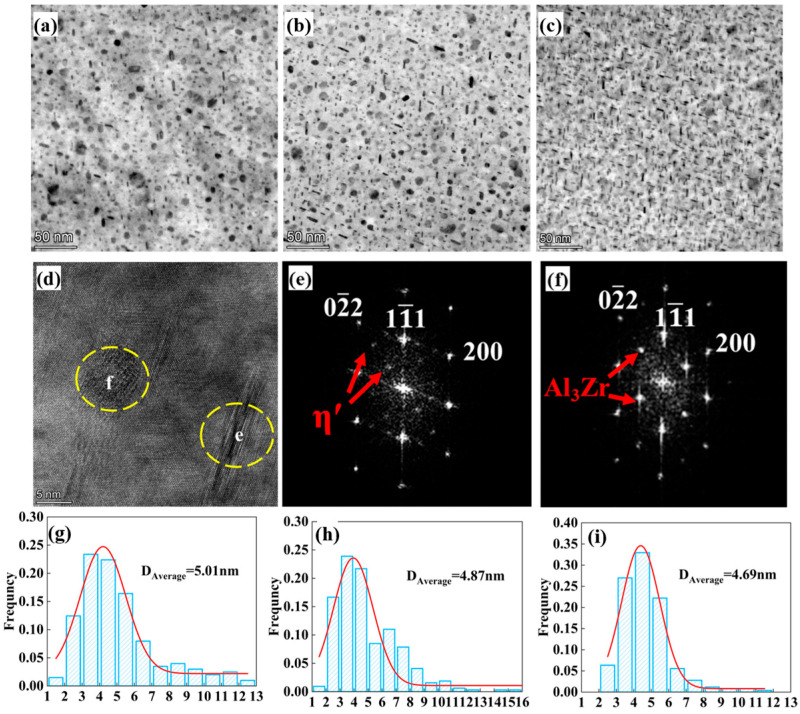
TEM images and precipitate diameter distribution along [110] Al direction after aging treatment: (**a**,**g**) Alloy 1; (**b**,**h**) Alloy 2; (**c**,**i**) Alloy 3. HRTEM image (**d**) and corresponding FFT images (**e**,**f**) are taken from Alloy 2.

The primary precipitates in Al–Zn–Mg–Cu alloys include the Guinier–Preston zone I (GPI), Guinier–Preston zone II (GPII), η′ phase, and η phase. To conduct a more in-depth examination of the impact of nanoscale precipitation on the characteristics of alloys, TEM analysis was performed on the three alloys. Figure 6 presents the BF images, showing that the precipitates formed in alloys with varying total (Zn + Mg) contents exhibit predominantly near-circular and rod-like morphologies. High-resolution transmission electron microscopy (HRTEM), combined with fast Fourier transform (FFT) analysis, identified the primary precipitates as η′ phases, while the larger particles were confirmed to be Al_3_Zr. Quantitative analysis using ImageJ 1.54 software revealed that the average sizes of precipitates were 5.01 nm, 4.87 nm, and 4.69 nm for the three alloys with increasing total (Zn + Mg) content, respectively.

**Figure 7:**
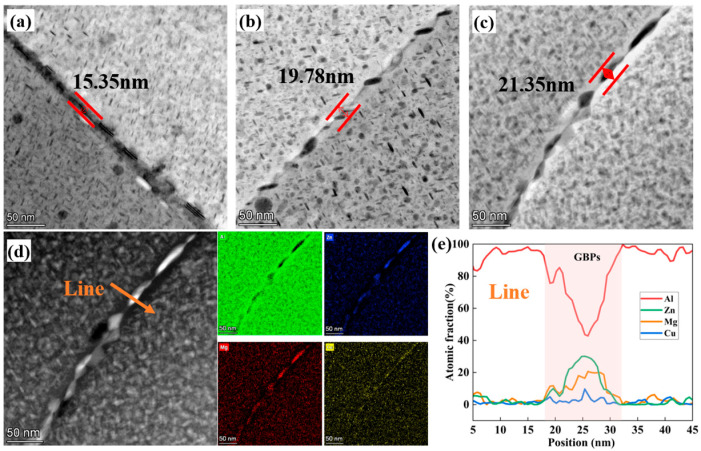
TEM images of grain boundary morphology after artificial aging treatment: (**a**) Alloy 1; (**b**) Alloy 2; (**c**) Alloy 3. (**d**) represents the EDS diagram at the grain boundary, taken from Alloy 3, while (**e**) is the composition distribution shown by the yellow line in (**d**).

Figure 7 shows the grain boundary morphologies of three alloys. It is evident that the size of the precipitates located at the grain boundaries increases with the total (Zn + Mg) content. Elevated total (Zn + Mg) content causes grain boundary precipitates (GBPs) to shift from discontinuous to continuous arrangements. Corresponding STEM-EDS analysis indicates that these GBPs are rich in Cu, Mg, and Zn and are typically identified as the η-MgZn2 phase. In addition, increasing the total (Zn + Mg) content promotes the broadening of the precipitation-free zones (PFZs) adjacent to the grain boundaries.

### 3.4. Mechanical Properties

**Figure 8:**
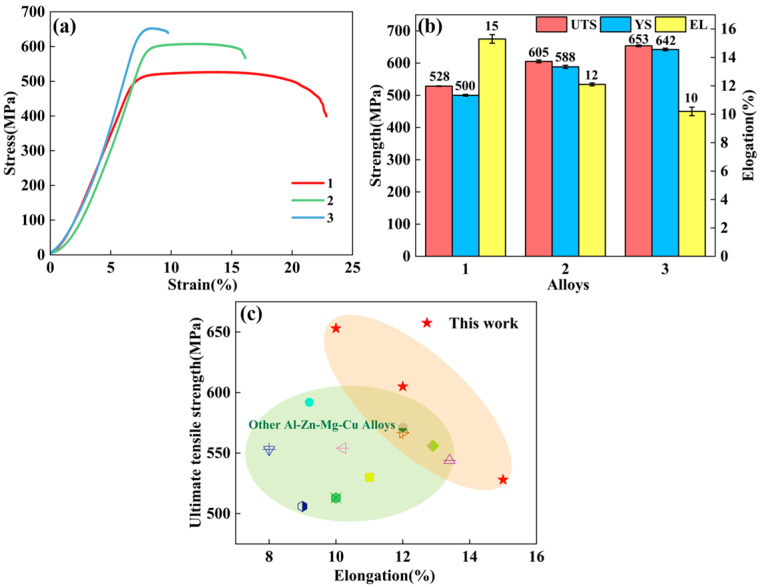
(**a**) Stress–strain curves; (**b**) tensile properties of three alloys in the T74 temper; (**c**) a comparison of the ultimate tensile strength and ductility of Al-Zn-Mg-Cu alloys [16,31,32,33,34,35,36,37,38,39].

The tensile properties of the aged alloys were evaluated to assess their macroscopic mechanical behavior. As shown in Figure 8, the mechanical characteristics of the three alloys are illustrated. Specifically, Figure 8a presents the engineering stress–strain curves, while Figure 8b displays the corresponding YS, UTS, and EL derived from these curves.

The results indicate that, under identical heat treatment conditions, increasing the total (Zn + Mg) content leads to a significant enhancement in both UTS and YS, which increase from 528 MPa and 500 MPa to 653 MPa and 642 MPa, respectively. However, this strength improvement is accompanied by a reduction in EL of approximately 33%. It can be seen that, compared with the other two alloys, Alloy 2 shows a higher strength while maintaining desirable plasticity, which may be attributed to the effect of the nested grain structure. Figure 8c presents the reported Ashby plots for Al–Zn–Mg–Cu alloys, depicting their tensile properties. Notably, the samples in this work exhibit a superior strength–ductility synergy compared to most Al–Zn–Mg–Cu alloys produced via similar processing routes.

### 3.5. Fracture Morphology

**Figure 9:**
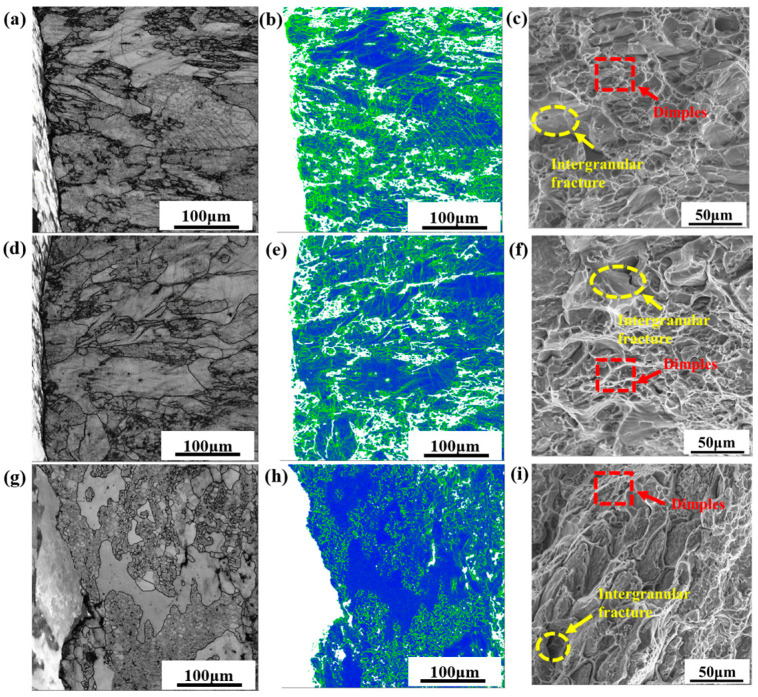
Band contrast (BC), KAM and SEM images of fracture microstructure: (**a**–**c**) Alloy 1; (**d**–**f**) Alloy 2; (**g**–**i**) Alloy 3.

Figure 9 illustrates the grain morphology and fracture characteristics across the crack path. Alloys 1 and 2 failed predominantly by transgranular fracture, with ductile dimples observed, whereas Alloy 3 exhibited mainly intergranular cracking along sub-grain boundaries. GND density maps (Figure 9b,e,h) reveal the variation in local dislocation orientation. Due to the significant plastic deformation of Alloys 1 and 2, strong dislocation motion is required to coordinate the deformation, which leads to an increase in GND density near the fracture. In contrast, the corresponding lower GND density in Alloy 3 is attributed to its limited plastic deformation. These findings indicate that the nested grain structure in Alloy 2 provides an advantageous balance between plastic deformability and strength.

### 3.6. Electrochemical Characteristics

**Figure 10:**
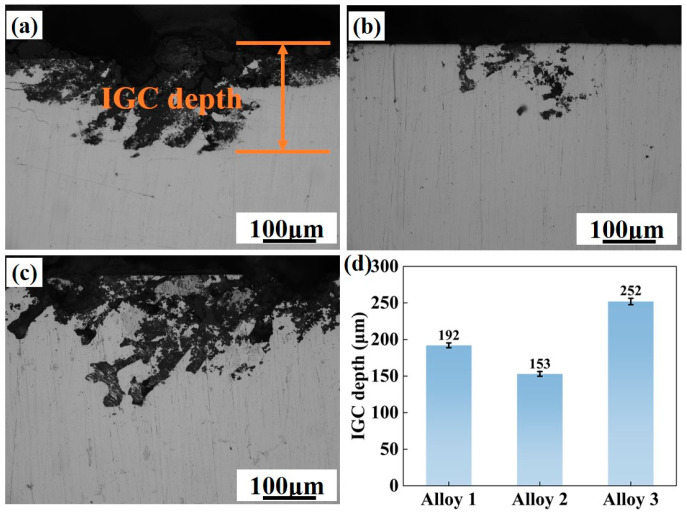
The intergranular corrosion image of the three alloys after aging treatment: (**a**) Alloy 1; (**b**) Alloy 2; (**c**) Alloy 3; (**d**) the measured IGC depth.

IGC testing was carried out on three different alloy compositions for assessing the impact of total (Zn + Mg) content on the IGC behavior of alloys. The results are presented in Figure 10. Among the three alloys, Alloy 2 exhibits the optimal IGC resistance, with a corrosion depth of 153 μm, followed by Alloy 1 at 196 μm. Alloy 3 shows the most severe IGC sensitivity, with a maximum corrosion depth of 258 μm, which indicates that increasing the total (Zn + Mg) content leads to a deterioration in intergranular corrosion resistance. In contrast, a moderate total (Zn + Mg) content around 10 wt.% appears to effectively reduce the susceptibility of the alloys to intergranular corrosion.

**Figure 11:**
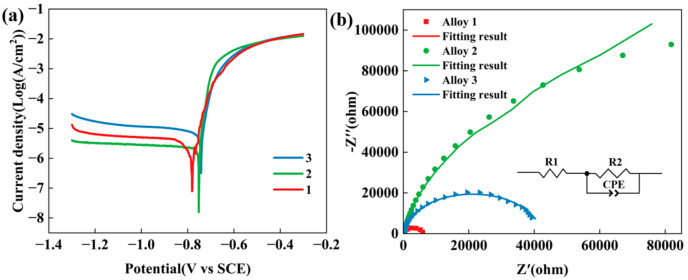
Electrochemical test results: (**a**) Tafel polarization curve; (**b**) impedance curve and equivalent circuit model.

Electrochemical tests are conducted on the three alloys, and the corresponding polarization curves are shown in Figure 11a. The curves exhibit similar overall trends, suggesting that the alloys undergo comparable corrosion mechanisms. Exceeding the corrosion potential (E_corr_) with applied potential leads to a marked increase in current density, resulting from the rupture of the protective oxide film on the alloy surface. Despite the similar shapes of the polarization curves, significant differences in both E_corr_ and corrosion current density (i_corr_) were observed among the alloys. It is well established that a more positive E_corr_ and a reduced i_corr_ indicate superior corrosion resistance [40,41]. In this study, E_corr_ and i_corr_ values were determined using the Tafel extrapolation method, and the results are summarized in Table 2. Notably, Alloy 2 exhibited an E_corr_ of −0.75 V, which is more positive than that of Alloy 1 (−0.78 V), and slightly less positive than that of Alloy 3 (−0.74 V). However, Alloy 2 showed a significantly lower i_corr_ value of 2.71 × 10^−6^ A·cm^−2^ compared to Alloy 3 (8.52 × 10^−6^ A·cm^−2^), indicating that Alloy 2—containing a total (Zn + Mg) content of 10.6 wt.%—possesses the best electrochemical corrosion resistance among the three.

The passivation film characteristics and alloy corrosion resistance were evaluated using Electrochemical Impedance Spectroscopy (EIS). Figure 11b displays the Nyquist plots and equivalent circuit model of the three alloys. Under the assumption of a similar corrosion mechanism, the electrochemical corrosion performance can be assessed by comparing the diameter of the capacitive arc in the high-frequency region of the Nyquist plot. A larger diameter denotes superior corrosion resistance [42,43]. Among the three alloys, Alloy 2 exhibits the largest arc radius, followed by Alloy 1, while Alloy 3 shows the smallest arc radius. This result further confirms that Alloy 2 possesses superior corrosion resistance compared to the other two alloys.

## 4. Discussion

### 4.1. The Effect of Mortise–Tenon Nested Grain Structure

During the MDF process, newly formed fine equiaxed grains nucleate and grow along the boundaries of the original coarse grains, resulting in a nested-like grain structure reminiscent of a mortise–tenon configuration (Figure 4b). This structure resembles a semi-rigid mortise–tenon joint, where the interlocking effect effectively constrains relative distortion between adjacent sub-grains. As a result, it enhances interfacial bonding strength and hinders dislocation movement, thereby significantly improving the overall structural strength of the material [15,44]. The formation of this nested structure can be attributed to variations in dislocation density and localized energy accumulation during multi-pass deformation, which partially promote dynamic recrystallization and lead to the development of a multi-scale grain structure comprising both micron- and submicron-sized grains.

In this study, the nested structure is mainly formed by the interaction of fine equiaxed grains and recrystallized grains or sub-grain interfaces. This structure not only achieves fine grain strengthening (Hall–Petch effect) but also forms a multi-scale grain structure. It can also carry out synergistic deformation to dissipate energy, increase the geometric obstruction of dislocation motion, and thus improve the structural strength of the material. In addition, the behavior of recrystallization—for example, the proportion of recrystallized grains in Alloy 2 reaches 51.8%—optimizes the randomness of grain orientation (Figure 4 and Figure 5). This reduction in texture intensity improves the material’s microstructural uniformity and reduces anisotropy during plastic deformation. Finally, the existence of this nested structure can not only ensure the strength increase caused by Zn and Mg alloying [21,25], but also maintain desirable plasticity.

### 4.2. The Effect of the Total Amount of (Zn + Mg) on the Mechanical Properties and Precipitated Phase

Age-hardenable Al–Zn–Mg–Cu alloys exhibit properties critically dependent on precipitate characteristics: size, density, and volume fraction. The primary strengthening precipitates comprise the GP zones, η′ phase, and η phase. The distribution and morphology of the η′ phase within the aluminum matrix critically influence the strengthening efficiency and corrosion resistance of the alloy [45,46].

Elevating the total (Zn + Mg) content from 9.6 wt.% to 11.7 wt.% significantly enhances UTS and YS (Figure 8), as evidenced by experimental data. Such mechanical improvement directly correlates with precipitated phase refinement, notably the η′ phase, where the average particle size drops from 5.01 nm to 4.69 nm. According to the Orowan strengthening mechanism, finer precipitates more effectively hinder dislocation motion, thereby contributing to the observed increase in mechanical strength [47]. It is worth noting that although the mechanical properties of Alloy 2 are lower than those of Alloy 3, its nested grain structure and narrow PFZs ensure structural stability. Therefore, Alloy 2 obtains excellent plasticity while maintaining high mechanical properties.

In order to further analyze the contributions of precipitates, grain structure, and dislocations to strength in detail, it is necessary to quantify the contribution of back stress to the strengthening effect. Al–Zn–Mg–Cu alloys derive their primary strengthening from four contributions: solid solution strengthening ($\sigma_{ss}$), dislocation strengthening ($\sigma_{Dis}$), grain boundary strengthening ($\sigma_{GB}$) and precipitation strengthening ($\sigma_{ppt}$). The corresponding theoretical yield strength ($\sigma_{Total}$) can be calculated using Formula (2) [48,49]:

(2) $$\sigma_{Total}=\sigma_{0}+\sigma_{GB}+\sigma_{ss}+\sigma_{Dis}+\sigma_{ppt}$$

where $\sigma_{0}$ is the stress of aluminum without texture, and the value is 10 MPa [50].$\;\sigma_{GB}$ refers to the blocking effect of grain boundaries on dislocations. A reduction in grain size leads to an increase in grain boundary density. This heightened density enhances the probability of dislocations interacting with grain boundaries during their motion. The resulting enhancement in grain boundary obstruction to dislocation movement contributes to higher alloy strength. The Hall–Petch equation is often used to quantitatively describe $\sigma_{GB}$ in experiments [51]:

(3) $$\sigma_{GB}=\sigma_{0}^{\prime }+k{\left(d\right)}^{-1/2}$$

where $\sigma_{0}^{\prime }$ is the lattice friction number of dislocation motion (16 MPa for aluminum), k is the Hall–Petch coefficient (12 MPa/$\sqrt{m}$ for Al–Zn–Mg–Cu alloy) [52], and d is the average grain diameter. For Alloy 1, Alloy 2, and Alloy 3, the respective average grain sizes are 139 μm, 120 μm, and 97 μm. The strengthening effect arising from grain boundaries is quantified in Table 3.

As the principal elements (Zn, Mg, Cu) dissolve into the aluminum matrix, this dissolution can cause lattice distortion, hindering dislocation movement and consequently improving both yield and tensile strength. However, the TEM micrograph in Figure 6 demonstrates that the aged alloy contains numerous nano-sized precipitates. This high density implies the precipitation of a substantial number of solute atoms from the matrix, which means most of the solvent atoms are consumed. Therefore, the $\sigma_{ss}$ can be reasonably ignored in the calculation of yield strength [47].

$\sigma_{Dis}$ describes a strengthening mechanism where the material’s inherent dislocations are utilized to obstruct subsequent dislocation motion, leading to enhanced strength. Dislocation strengthening can be calculated according to the Taylor equation [53,54]:

(4) $$\sigma_{Dis}=M\alpha Gb\rho^{\frac{1}{2}}$$

where M = 3.06 represents the Taylor factor, α is a constant (~0.24) [47], ρ is the dislocation density, G = 26.9 GPa is the shear modulus, and b = 0.286 nm is the Burgers vector of aluminum. The results are shown in Table 4.

Aging precipitation strengthening is a key mechanism for enhancing alloy strength by impeding dislocation motion through the controlled formation, size, distribution, and volume fraction of second-phase precipitates. During aging, fine and uniformly distributed nanoscale precipitates form from the supersaturated solid solution. These particles interact with moving dislocations, thereby significantly increasing the strength and hardness of the alloy.

Based on Figure 6, η’ and Al_3_Zr are the predominant aging precipitates across all samples, which implies that the dislocation bypass mechanism is principally responsible for strengthening [55]. The Orowan model provides a formula for estimating the corresponding yield strength increase [56]:

(5) $$\sigma_{ppt}=M\frac{0.4Gb}{\pi \sqrt{1-v}}\frac{\ln\left(2\sqrt{2/3}r/b\right)}{2\bar{r}\left(\sqrt{\pi /4f}-1\right)}$$

where $\bar{r}=\sqrt{\frac{2}{3}}r$ represents the average radius of aging precipitates, f represents volume fraction, and v = 0.33 represents Poisson’s ratio. f is calculated by the method described in reference [57]. The calculation results are shown in Table 5.

**Figure 12:**
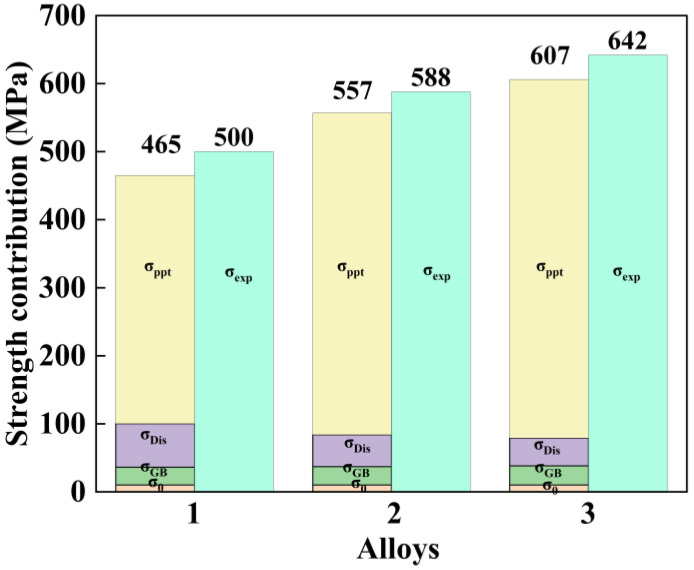
The comparison between the theoretical calculation and the experimental measured value of $\sigma_{Total}$.

Figure 12 presents a summary of the distinct contributions made by various strengthening mechanisms across the three alloys under investigation. The calculated yield strength values are consistently lower than the experimentally measured results, indicating the presence of additional strengthening contributions not fully captured by conventional models. This discrepancy suggests that complex interactions among the different strengthening mechanisms, such as dislocation–precipitate interactions, grain boundary effects, and texture, may contribute synergistically to the overall mechanical performance and cannot be entirely isolated or quantified through simplified theoretical calculations [58]. Although the calculated models might not account for every enhancement mechanism, analysis reveals that strengthening from dislocation and precipitation hardening principally drive the improved mechanical performance observed in all three alloys.

### 4.3. Corrosion Resistance

The results demonstrate that the corrosion sensitivity of Al–Zn–Mg–Cu alloys is closely correlated with their microstructure. On the one hand, the continuous η-MgZn_2_ precipitates lining grain boundaries act as anodic phases, forming galvanic couples with the matrix or PFZs and thereby accelerating localized anodic dissolution. On the other hand, the solute-depleted PFZs possess a lower electrochemical potential, which further exacerbates the local corrosion rate [59]. During electrochemical corrosion, the stability of the surface oxide film is critical. Corrosive ions such as Cl^−^ tend to adsorb at film defects, initiating pitting corrosion that propagates along grain boundaries or precipitate/matrix interfaces, ultimately leading to localized exfoliation or overall structural instability [60,61].

**Figure 13:**
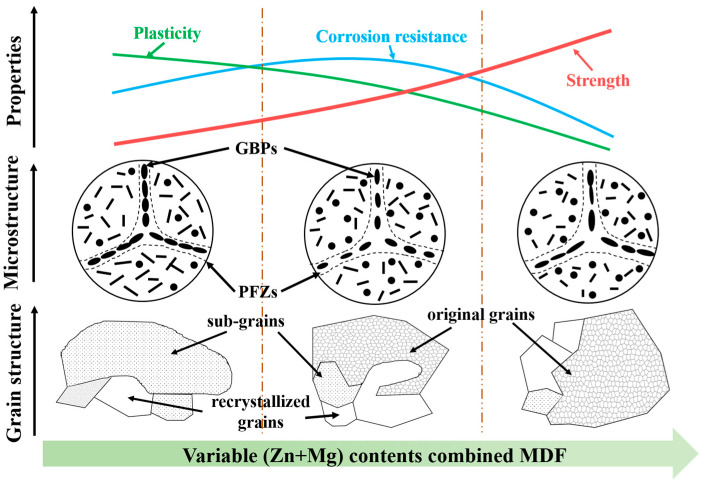
A schematic diagram of the microstructure and property evolution of alloys with different total (Zn + Mg) contents during MDF process.

The experimental results indicate that Alloy 2 exhibits the best corrosion resistance, as evidenced by its lowest corrosion current density (i_corr_ = 2.71 × 10^−6^ A·cm^−2^). Additionally, the intergranular corrosion depth of Alloy 2 (153 μm) is significantly lower than that of Alloy 1 (196 μm) and Alloy 3 (258 μm). EIS analysis further supports this observation, with the impedance parameters following the trend. This superior corrosion resistance in Alloy 2 (Figure 7) stems primarily from its discontinuous grain boundary precipitates. These features act as barriers to corrosive media penetration and diminish the galvanic coupling effect at grain boundaries relative to the matrix, consequently lowering susceptibility to intergranular corrosion [62]. Research demonstrates a sufficiently large potential difference between PFZs and GBPs, providing key electrochemical conditions for IGC [24,63,64]. Exceeding critical PFZ width degrades the corrosion resistance of alloys, since broadened PFZs become anodically active versus the matrix, amplifying potential disparity [65,66,67]. Hence, the PFZ dimension critically governs the corrosion performance of Alloy 2. The measured PFZ widths for Alloy 1, Alloy 2, and Alloy 3 are 15.35 nm, 19.78 nm, and 21.35 nm, respectively. A schematic diagram of the evolution of the microstructure and properties of different total (Zn + Mg) contents during the MDF process is shown in Figure 13.

## 5. Conclusions

In this work, we examined the precipitation behavior of alloys along, with its influence on the mechanical properties and corrosion resistance of different total (Zn + Mg) content Al–Zn–Mg–Cu alloys during MDF. The conclusions can be summarized as follows:

(1) MDF enables the alloys to obtain a mortise and tenon nested grain structure that suppresses grain boundary slip and strengthens interfaces via mechanical interlocking. Simultaneously, the synergistic deformation between nested submicron and micron grains significantly impedes dislocation motion.
(2) Increasing total (Zn + Mg) content enlarges grain boundary PFZs and transitions the η phase from discontinuous to continuous distribution. Electrochemical testing reveals that this elevation diminishes corrosion resistance. However, Al-8.6Zn-1.55Mg-1.9Cu-0.11Zr (Zn + Mg = 10.15 wt.%) exhibits superior electrochemical stability, evidenced by its minimal corrosion current density. IGC testing confirms that this composition provides relatively desirable corrosion resistance.
(3) Elevated total (Zn + Mg) content strengthens alloys through η′ precipitate refinement. Al-9.6Zn-2.1Mg-1.9Cu-0.11Zr (Zn + Mg = 11.7 wt.%) has continuous η–MgZn_2_ at grain boundaries and widened PFZs induce intergranular corrosion. Al-8.6Zn-1.55Mg-1.9Cu-0.11Zr (Zn + Mg = 10.15 wt.%) optimally balances precipitation strengthening and grain boundary refinement, enhancing both strength and corrosion resistance.

## Figures and Tables

**Table 1:** The precise composition of the alloys under investigation (wt.%).

Alloys	Zn	Mg	Cu	Zr	Al	(Zn + Mg)
Alloy 1	8.6	1	1.9	0.11	Bal.	9.6
Alloy 2	8.6	1.55	1.9	0.11	Bal.	10.15
Alloy 3	9.6	2.1	1.9	0.11	Bal.	11.7

**Table 2:** Electrochemical parameters.

Alloys	E_corr_ (V)	i_corr_ (A·cm^−2^)	R1 (Ω·cm^2^)	R2 (Ω·cm^2^)	CPE (SS^n^ cm^−2^)
Alloy 1	−0.78	2.93 × 10^−6^	30.11 ± 0.36	6087 ± 97	5.16 × 10^−6^ ± 0.14 × 10^−6^
Alloy 2	−0.75	2.71 × 10^−6^	67.43 ± 1.01	226,140 ± 14,986	9.34 × 10^−6^ ± 0.18 × 10^−6^
Alloy 3	−0.74	8.52 × 10^−6^	29.38 ± 0.22	42,225 ± 500	6.71 × 10^−6^ ± 0.081 × 10^−6^

**Table 3:** Calculated contributions from grain boundaries to alloy strengthening.

Sample	$\sigma_{Gb}$/MPa
Alloy 1	26.2
Alloy 2	27.0
Alloy 3	28.2

**Table 4:** Dislocation strengthening calculation results.

Sample	Average ρ/10^14^ m^−2^	$\sigma_{Dis}$/MPa
Alloy 1	1.29	64
Alloy 2	0.70	47
Alloy 3	0.55	41

**Table 5:** Calculated value of aging precipitation strengthening.

Sample	$\bar{r}$/nm	f/%	$\sigma_{ppt}$
Alloy 1	2.04	1.4	365
Alloy 2	1.99	2.1	473
Alloy 3	1.91	2.4	527

## Data Availability

The raw/processed data required to reproduce these findings cannot be shared at this time as the data also forms part of an ongoing study.
